# Ultrasound assisted wall-breaking extraction and primary structures, bioactivities, rheological properties of novel *Exidia yadongensis* polysaccharide

**DOI:** 10.1016/j.ultsonch.2023.106643

**Published:** 2023-10-30

**Authors:** Ying Tang, Yuzhi Miao, Min Tan, Qinqin Ma, Chengyi Liu, Mei Yang, Yanqiu Su, Qi Li

**Affiliations:** a Key Laboratory of Land Resources Evaluation and Monitoring in Southwest, Ministry Education of China, Chengdu, Sichuan 610066, China; b College of Life Sciences, Sichuan Normal University, Chengdu, Sichuan 610066, China; c PanZhiHua City Academy of Agricultural and Forestry Sciences, Panzhihua, Sichuan 617061, China

**Keywords:** *Exidia yadongensis* polysaccharide, Ultrasound extraction, Response surface, Primary structure, Bioactivities, Rheological properties

## Abstract

New natural multifunctional polysaccharide and its innovatory extraction technology may be urgently needed for food industries. Our aims were to establish new extraction method and investigate the primary structures, bioactivities and rheological properties of novel *E. yadongensis* polysaccharide (EYP). Ultrasound assisted mechanical wall-breaking extraction (MAUE) was successfully established for the EYP extraction from a new *E. yadongensis*. Based on the MAUE with RSM, the polysaccharide yield of 17.92 ± 0.56 % with the optimal parameters of five extraction factors were obtained, and current MAUE was characterized by its high yield, low extraction temperature and short ultrasound time. After the isolation and purification, the EYP as a protein-bound polysaccharide was obtained. FT-IR and NMR analysis showed that the main backbone of the EYP comprised of (1 → 4)-*β*-D-glucopyranosyl and (1 → 6)-*ɑ*-D-mannopyranosyl groups; EYP exhibited significant antioxidant, antibacterial, antitumor, antidiabetic activities, and good viscoelastic properties in low pH solutions (*P* < 0.05). The EYP may be used as a natural functional and cohesive agent in food industries.

## Highlights

• MAUE was established by low temperature and short time, making it perfect for the extraction of heat-labile polysaccharide.
• The main backbone of the EYP comprised of (1 → 4)-β-D-glucopyranosyl and (1 → 6)-ɑ-D-mannopyranosyl groups.
• EYP from *E. yadongensis* exhibited significant antioxidant, antibacterial, antitumor, and antidiabetic activities.
• EYP solutions from *E. yadongensis* exhibited good viscoelastic properties and tolerance in low pH conditions.

## 1 Introduction

Mushroom polysaccharide, as one of the most important bioactive compounds, possess antioxidant, antibacterial, antidiabetic, antiinflammatory and antitumor activities [1], [2], and has also exhibited remarkable ability to act as an emulsifier [3]. Thus, mushroom polysaccharide has received increasing demands as a prospective natural additive in the nutraceutical and pharmaceutical industries [4]. To date, number of researches relating to extraction technologies, structures, bioactivities, rheological properties and applications of polysaccharide from numerous medical and edible mushrooms are being reported [5], [6], [7].

*Exidia yadongensis*, as a unique and precious edible mushroom species named according to the methods of morphological examination and phylogenetic analyses, is only found in Yadong county of Tibet, China. Nowadays, a large scale of *E. yadongensis* is being widely cultivated in her native area [8]. However, the effective application of *E. yadongensis* is an urgent problem to be solved. The majority of *E. yadongensis*’s chemical components are polysaccharides, all eight essential amino acids, gelatin and different terpenes, which may play key roles in the maintenance of physiological functions such as lipid metabolism, blood sugar balance and protein synthesis and decomposition [9]. So far, the study on *E. yadongensis* polysaccharide (EYP) has not been reported. Therefore, an investigation of EYP including extraction methods, primary structures, bioactivities and rheological properties is valuable and necessary.

The different extraction technologies of polysaccharide may result in different yields, primary structures and functional properties [10], [11], [12]. So far, the hot water extraction (HWE), as one of the simplest and earliest leaching method methods, is often used to extract water-soluble polysaccharide from natural materials [13], [14]. However, HWE can only extract a part of plant-derived polysaccharides, number of cell polysaccharides (CPS) can’t be obtained [15]. To address these the shortcomings, many innovative methods, such as ultrasound-enzyme assisted extraction (UEAE), ultrasonic-microwave-assisted extraction (UMAE), and so on, have been developed to extract CWPS from different plant and mushroom materials, which can break through the cell wall of materials to promote the polysaccharide into the solution, and the yield of polysaccharide was significantly increased [5], [16], [17]. Nonetheless, these techniques basically needed to undergo a battery of treatments, which not only increase equipment but also may impact on the primary structures, bioactivities and rheological properties of the polysaccharides [18].

Recent research revealed that the cell-wall breaking extraction by mechanical shearing can improve both the yields and bioactivities of the polysaccharide in the fruiting body of *Ganoderma lucidum* and spores [19]. Moreover, compared to single extraction technology applied, combination technologies have more influences on the cell wall disruption of plant materials [20]. Therefore, it is very meaningful to establish a novel method of mechanical wall-breaking assisted ultrasound extraction (MAUE) that can be been utilized to extract the EYP and other plant-derived polysaccharide, and the primary structures, bioactivities, rheological properties of the EYP obtained by MAUE method are worth further research.

## 2 Materials and methods

### 2.1 Materials and reagents

Freeze-dried *E. yadongensis* was obtained from Yadong county of Tibet, China. Methanol and chloroform (GC–MS grade) were obtained from Sigma Aldrich Company (USA). All other reagents (Analytical grade) were obtained from Changzhen Reagents Co. Ltd (Chengdu, China).

### 2.2 Mechanical wall-breaking equipment

Model TY-8L food shearing disintegrator (Jinan Tianyu special equipment Co. Ltd., China) is used to break the cell-walls of the freeze-dried *E. yadongensis.* The operating temperature of this equipment is from 0 to −40 °C. In this study, this equipment was used to replace the traditional powder machine.

### 2.3 EYP extraction

#### 2.3.1 Extraction process of MAUE

Freeze-dried *E. yadongensis* were crushed into broken powder using cell-wall broken technology by food shearing disintegrator at −30 ± 2 °C, and the powders with different cell-wall broken rates (WBR) was obtained. The powders were defatted with 95 % ethanol solution, and dried for 24.0 h at 40.0 °C. Then, EYP was extracted by the ultrasonic processor (KQ-400KDB, Kunshan instrument Co. Ltd., China) in different temperature water. The extracts were filtered and concentrated, and the free proteins of crude EYP were removed using the sevage method. The deproteinized solution was centrifuged at 5000 rpm for 20.0 min and freeze-dried as water-soluble crude EYP. EYP yield (%) was obtained according to Eq. (1):

(1) $$Y_{\mathrm{EYP}}\left(\%,W/W\right)=M_{\mathrm{weightofextracts}}/M_{\mathrm{weightofdriedmaterial}}\times 100\%$$

#### 2.3.2 MAUE optimization design

Single-factor optimization under different WBR: according to the results of pre-experiment, the WBR of *E. yadongensis* was set at 70.0, 80.0 and 90.0 %, respectively. The effect of water to wall broken powder ratio (WPR, 55.0–80.0 mL/g, 6 gradients), ultrasonic power (UP, 200.0–400.0 W, 6 gradients), ultrasonic time (UT, 10.0–60.0 min, 8 s off, 3 s on, 6 gradients) and extraction temperature (ET, 25.0–50.0 °C, 6 gradients) on the yield of the EYP were studied.

RSM optimization of combined WBR: based on the results of single-factor tests, a central composite design (CCD) with four factors including WPR (X_1_), UP (X_2_), UT (X_3_) and ET (X_4_) were coded at five levels (-2, −1, 0, 1, 2) on the EYP yield (Table 1). According to the Design Expert software (V11.1.2.0, USA), the experimental data were obtained, and predicted response values were analyzed.

**Table 1:** All strains tested for antibacterial activities.

No.		Strains	ATCC
1	Gram-negative	*Salmonella enteritidis*	13,076
2		*Escherichia coli*	25,922
3		*Shigella sonnei*	29,930
4		*Yersinia enterocolitica*	27,729
5		*Pseudomonas aeruginosa*	27,853
6	Gram-positive	*Staphylococcus aureus*	29,213
7		*Enterococcus faecalis*	29,212
8		*Bacillus cereus*	10,876
9		*Listeria monocytogenes*	19,115

### 2.4 EYP preparation and purification

Using the optimal extraction conditions, 100 g of EYP was obtained by the MAUE, ethanol precipitation (75 %, v/v), dialysis, and freeze-dried. The total sugar and protein contents of EYP was measured according to the description of He et al. [21].

Purification of the EYP were conducted by the description of Qian et al. [22] with minor modification. Briefly, the EYP (200.0 mg) was dissolved in 20.0 mL distilled water. The mixed solution was loaded onto the Q-Sepharose FF column, and eluted with and NaCl solutions of 0.1, 0.3 and 0.5 mmol/mL and distilled water at a flow rate of 60.0 mL/h, respectively. The eluents of 5.0 mL/tube were collected using automatic fraction collector. Then, the fractions were purified by a Sephadex G-100 gel column, respectively. Gel column was eluted with NaCl solution of 0.1 mol/L at a flow velocity of 20.0 mL/h. The eluents (5.0 mL/tube) were collected using automatic fraction collector, dialysed, and freeze-dried for further analyses. UV–Vis spectrophotometer (UV-2401PC) was used to scan the EYP solution (6 mg/mL) for proteins and nucleic acid traces in the range of 200–800 nm, respectively.

### 2.5 EYP characterization analysis

#### 2.5.1 Chemical compontents

The total polysaccharides content of EYP was measured using D-glucose as standard by the description of Qian et al. [22]. Protein content of EYP was determined by Bradford’s assay [21]. The Ultraviolet visible (UV–Vis) was recorded with a spectrophotometer (UV-2401PC) in the range of 200–800 nm.

#### 2.5.2 Molecular weight

Number-average molecular weight (*M_n_*) and weight-average molecular (*M_w_*) of the EYP were texted using HPGPC according to the method of Qian et al. [22], combined with multi-angle laser light scattering, and the *M_n_* and *M_w_* were determined by the calibration curves obtained according to different molecular weight dextran standards.

#### 2.5.3 Monosaccharide analysis of EYP

The monosaccharide composition of the EYP were determined by a method of Khaskheli et al. [23]. The monosaccharide was analyzed with GC–MS (Shimadzu Corporation, Japan).

#### 2.5.4 FT–IR and NMR assay

The EYP (1.0 mg) as ground with KBr (100.0 mg) and pressed into pellets for assay. Spectroscopy was recorded on a spectrum one fourier transform infrared spectrometer (FT–IR) with a range from 500 to 4000 cm^−1^.

For nuclear magnetic resonance (NMR) measurements, 20.0 mg of the dried EYP was exchanged with deuterium several times by freeze-dried in D_2_O, and then dissolved in 1.0 mL D_2_O (99.97 %) for 6.0 h. NMR spectra (^13^C NMR and ^1^H NMR) of the EYP was recorded by a NMR spectrometer (Bruker Avance III-800 MHz, Switzerland) with chemical shifts given in proper ppm.

### 2.6 EYP bioactivities analysis

#### 2.6.1 Antioxidant activities

The DPPH• and •OH scavenging capacity of the EYP were evaluated as described by Du et al. [24]. The scavenging rate of the superoxide radical and the reducing power of the EYP was evaluated as described by Sheng et al. [6].

#### 2.6.2 In vitro antibacterial activities

In this study, *in vitro* antibacterial activities, minimum inhibitory concentrations (MIC) and minimum bactericidal concentrations (MBC) against several food-borne pathogens were evaluated by using Gram-negative (G^-^）and Gram-positive (G^+^) bacterial species (Table 1). Antibacterial activities of the EYP were assessed to all strains by the reported method of Wang, Lu, Wu, and Lv [25]. Briefly, each inoculum (0.1 mL) containing 1 × 10^7^ CFU /mL was spread on nutrient agar plate, and 0.2 mL of different concentrations EYP (1.0, 2.0, 5.0, 10.0, 15.0 and 20.0 mg/mL) were injected into Oxford cups (6.0 mm in diameter), respectively. The diameter of inhibition zone (DIZ) was determined at 37.0 °C after 24.0 h. MIC and MBC of the EYP were measured according to broth microdilution method [26].

#### 2.6.3 Antidiabetic and antitumor activities

Antidiabetic activities of the EYP was measured by using the inhibitory rate of EYP against α-amylase and α-glucosidase; and antitumor activities against of the EYP were evaluated by using the inhibitory rate of EYP against colon (Caco-2) and breast (MCF-7) cancer cells were texted according to the described method of Ayyash et al. [27]. The concentrations of the texted EYP were in the range of 50.0–300.0 μg/mL and 5.0–30.0 mg/mL, respectively.

### 2.7 Rheological properties assay

To evaluate the rheological properties of EYP, different solutions (3 and 6 %) were prepared by dissolving the EYP powder in ultrapure H_2_O at pH 6.0 and 4.0, respectively. The rheological tests of EYP solutions were performed using a 50 mm diameter DHR-2 rheometer of the TA Instruments Company from USA (1° cone angle, 50 µm gap) at 25 ± 0.1 °C with 0.1 – 100 s^−1^ shear rates. The apparent viscosity (η) and shear stress (τ) of the EYP solutions were assessed by the previous reported method [27]. The power law models were used to describe the flow curves of EYP as Eq. (2):

(2) $$\tau =K{\dot{\gamma }}^{n}$$

where τ, k, ●γ and n is represented shear stress (Pa), consistency coefficient, shear rate (s^−1^) and flow behavior index, respectively.

The energy storage modulus (G′) and the loss modulus (G′′) of EYP solutions were estimated by the frequency sweep test with DHR-1 rheometer (40 mm diameter). The scanning frequency were established in a strain from 0.1 to 100 s^−1^ at 25 ± 0.1 °C. The data were fitted as Eq. (3):

(3) $$G^{\prime }=m{\left(\omega \right)}^{n}$$

where G′, ω, m and n are represented the storage modulus, angular frequency, model constants and the slope, respectively.

### 2.8 Statistical analysis

All experiment were determined in triplicates, and values are represented as the means ± SD. Statistical analysis software SAS v9.2 was used to carry out all the statistical analyses. Student’s test was carried out for mean comparisons at a significant level of *P* < 0.05. Letters (a - q) different in the same figures or table differ statistically.

## 3 Results and discussion

### 3.1 Effects of four factors under different WBR on EYP yield

**Fig. 1:**
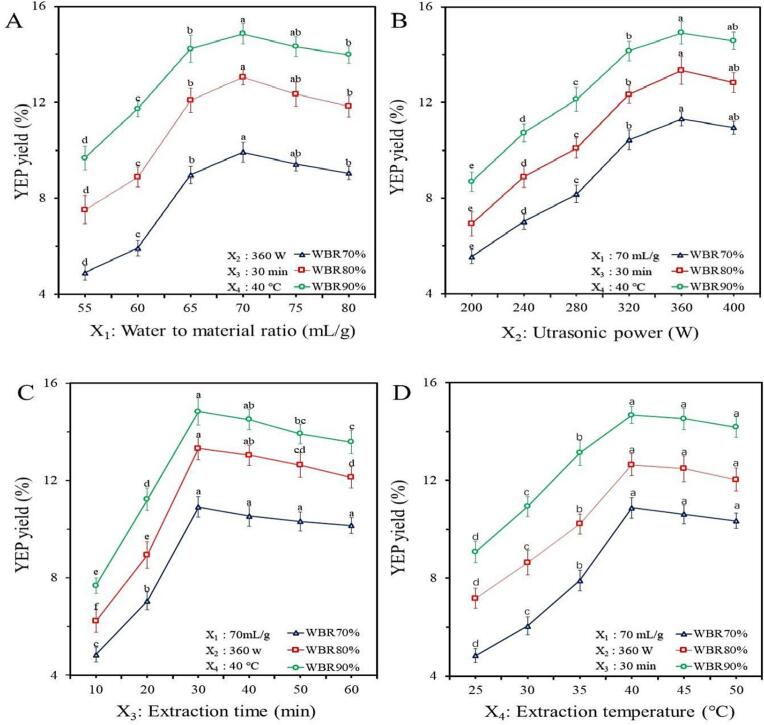
Effects of WPR (X_1_), UP (X_2_), UT (X_3_) and ET (X_4_) on the EYP yield under different WBR by single factor experiment for optimization of EYP extraction (A - D) from *E. yadongensis.*

Under different WBR, the effects of WPR (X_1_), UP (X_2_), UT (X_3_) and ET (X_4_) on the EYP yield are showed in Fig. 1(A - D). Fig. 1A - D indicated that the EYP yield increased obviously along with an increase of WBR from 70.0 % to 90.0 % (*P* < 0.05) and all highest yield was obtained at WBR 90.0 %, which could be explained that the cell wall breakage improved the EYP dissolution from *E. yadongensis*, and the EYP solubility increased with the increase of the WBR.

Fig. 1A showed that the EYP yield increased significantly along with an increase of WPR from 55.0 to 70.0 mL/g (*P* < 0.05). When X_1_ increased further, the EYP yield decreased rapidly (*P* < 0.05). The highest yield of the EYP was obtained at 70.0 mL/g. The WPR was significantly bigger than that obtained by other ultrasonic extraction [28], which can be explained that ultrasonic cavitation effect required the larger concentration differences to improve the mass transfer rate between internal cells and external solvents [29].

Fig. 1B indicated that the EYP yield increased obviously with the improvement of UP from 200.0 to 360.0 W (*P* < 0.05), and after that, there was a slight decline in the yield of the EYP. Similarly, the UP of ultrasound assisted wall-breaking extraction was obviously higher than that obtained by other ultrasonic extraction, which may explain the greater ultrasonic power can promote the transfer of intracellular molecular of the broken cell-wall powder into solvents, and too big ultrasonic power may cause the degradation of the polysaccharides [30].

Fig. 1C showed that the yield of the increased quickly (*P* < 0.05) with UT from 10.0 to 30.0 min, and the yield decreased when prolonging time. In this study, a relative short ultrasonic extraction time was obtained, which may explain that the effects of ultrasonic cavitation increased the diffusion rates of intracellular ingredient and the solvent, and resulting in promoted extraction efficiency under the condition of high WPR and strong UP [31].

Fig. 1D showed that the yield increased obviously (*P* < 0.05) with increasing ET from 25.0 to 40.0 °C. After 40.0 °C, the yield began to decline slightly. These results indicated that enhancing the temperature would improve the rate of diffusion and increased the release of the EYP in the liquid [32]. Surprisingly, the ET was obviously lower than that reported by other ultrasonic extraction [28], which showed that ultrasound assisted wall-breaking extraction can significantly reduce the dissolution temperature. Therefore, 70.0 mL/g ratio, 360.0 W power, 30.0 min time and 40.0 °C temperature with the WBR 90.0 % were determined as central point for further experiment.

### 3.2 Response surface optimization results of MAUE

#### 3.2.1 Model fitting and optimized analysis

According to single-factor experimental result, the influence of the interaction of four factors on the yield of EYP was obtained through 30 runs (Table 2). Table 2 indicated the experimental values of 30 runs. The data obtained by the CCD experiment was analyzed by using Design-Expert V11.1.2.0 under the analysis of variance (ANOVA), and the quadratic regression equation between four independent variables and predicted yield was showed as Eq. (4):

(4) $$\mathrm{Yield}=16.95+0.77X_{1}+0.91X_{2}+1.78X_{3}+0.69X_{4}+0.04X_{1}X_{2}-0.52X_{1}X_{3}+0.18X_{1}X_{4}-0.02X_{2}X_{3}+0.05X_{2}X_{4}+0.17X_{3}X_{4}-1.59X_{1}^{2}-1.28X_{2}^{2}-1.85X_{3}^{2}-1.44X_{4}^{2}$$

**Table 2:** CCD design of four variables with the actual and predicted values for EYP yield.

Run	Coded (actual) variables				Response (Extraction yield, %)	
	X_1_ (mL/g)	X_2_ (W)	X_3_ (min)	X_4_ (°C)	*Actual	Predicted
1	−1 (65.0)	−1 (320.0)	−1 (20.0)	−1（35.0）	6.82 ± 0.47^q^	6.62
2	1 (75.0)	−1	−1	−1	8.31 ± 0.11^n^	8.68
3	−1	1 (400.0)	−1	−1	7.36 ± 0.10^o^	7.56
4	1	1	−1	−1	9.32 ± 0.23 ^l^	9.93
5	−1	−1	1 (40.0)	−1	11.27 ± 0.52^i^	10.74
6	1	−1	1	−1	10.66 ± 0.47^j^	10.90
7	−1	1	1	−1	11.78 ± 0.12 ^h^	12.44
8	1	1	1	−1	12.63 ± 0.46^f^	12.66
9	−1	−1	−1	1 (45.0)	6.69 ± 0.21^q^	7.12
10	1	−1	−1	1	10.50 ± 0.00^j^	9.98
11	−1	1	−1	1	9.06 ± 0.11 ^k^	9.00
12	1	1	−1	1	11.14 ± 0.13^i^	12.02
13	−1	−1	1	1	13.16 ± 0.17^e^	13.01
14	1	−1	1	1	13.28 ± 0.60^e^	12.88
15	−1	1	1	1	13.78 ± 0.00^d^	13.90
16	1	1	1	1	14.41 ± 0.59^c^	14.84
17	−2 (60.0)	0 (360.0)	0 (30.0)	0 (40.0)	8.88 ± 0.1 ^m^	9.05
18	2 (80.0)	0	0	0	12.99 ± 0.09^e^	12.13
19	0 (70.0)	−2 (280.0)	0	0	8.89 ± 0.12 ^m^	10.01
20	0	2 (440.0)	0	0	15.44 ± 0.52^b^	13.65
21	0	0	−2 (10.0)	0	7.12 ± 0.49^p^	5.99
22	0	0	2 (50.0)	0	12.62 ± 0.59^f^	13.11
23	0	0	0	−2 (30.0)	10.83 ± 0.03^j^	9.91
24	0	0	0	2 (50.0)	12.22 ± 0.59 ^g^	12.57
25	0	0	0	0	16.92 ± 0.57^a^	16.95
26	0	0	0	0	16.84 ± 0.43^a^	16.95
27	0	0	0	0	17.22 ± 0.39^a^	16.95
28	0	0	0	0	16.91 ± 0.53^a^	16.95
29	0	0	0	0	17.14 ± 0.30^a^	16.95
30	0	0	0	0	16.97 ± 0.34^a^	16.95

Based on ANOVA, adequacy and fitness of the quadratic regression model were showed in Table 2. Table 2 showed that the model F-value of 24.59 was extreme significant (*P* < 0.0001). On the basis of the *F*-test, the coefficient estimates for the optimization showed that the linear coefficients and quadratic coefficients of all independent variables, and cross coefficients (X_1_X_3_) significantly influenced on the extraction yield (*P* < 0.05), and other model terms are not significant. The lack of fit *F*-value of 244.52 was tremendously significant relative to the pure error (*P* < 0.0001). Table 3 showed that the predicted R^2^ of 0.7598 was in reasonable agreement with the R^2^_Adj_ of 0.9193. Ratio of 16.22 indicated an adequate signal, and C.V. % of 7.95 showed that a good reliability of the experiment precision.

**Table 3:** Significance test for regression coefficient by apply ANOVA.

Sources	Sum of squares	DF	Mean square	*F*-value	*P*-value	aSignificant
Model	314.86	14	22.49	24.59	< 0.0001	***
X_1_	14.34	1	14.34	15.67	0.0013	**
X_2_	19.97	1	19.97	21.83	0.0003	**
X_3_	76.22	1	76.22	83.32	<0.0001	***
X_4_	11.55	1	11.55	12.63	0.0029	**
X_1_X_2_	0.0315	1	0.0315	0.0344	0.8553	
X_1_X_3_	4.36	1	4.36	4.76	0.0454	*
X_1_X_4_	0.5439	1	0.5439	0.5946	0.4526	
X_2_X_3_	0.0068	1	0.0068	0.0074	0.9324	
X_2_X_4_	0.0333	1	0.0333	0.0364	0.8512	
X_3_X_4_	0.4590	1	0.4590	0.5018	0.4896	
X_1_^2^	68.92	1	68.92	75.34	<0.0001	***
X_2_^2^	44.77	1	44.77	48.94	<0.0001	***
X_3_^2^	94.01	1	94.01	102.78	<0.0001	***
X_4_^2^	56.69	1	56.69	61.97	<0.0001	***
Residual	13.72	15	0.9147			
Lack of Fit	13.69	10	1.37	244.52	<0.0001	***
Pure Error	0.0280	5	0.0056			
Cor Total	328.58	29				

a *R^2^* = 0.9582, *R^2^_Adj_* = 0.9193, C.V. % = 7.95, * (*P* < 0.05); ** (*P* < 0.01); *** (*P* < 0.001).

#### 3.2.2 Response surfaces analysis of EYP

**Fig. 2:**
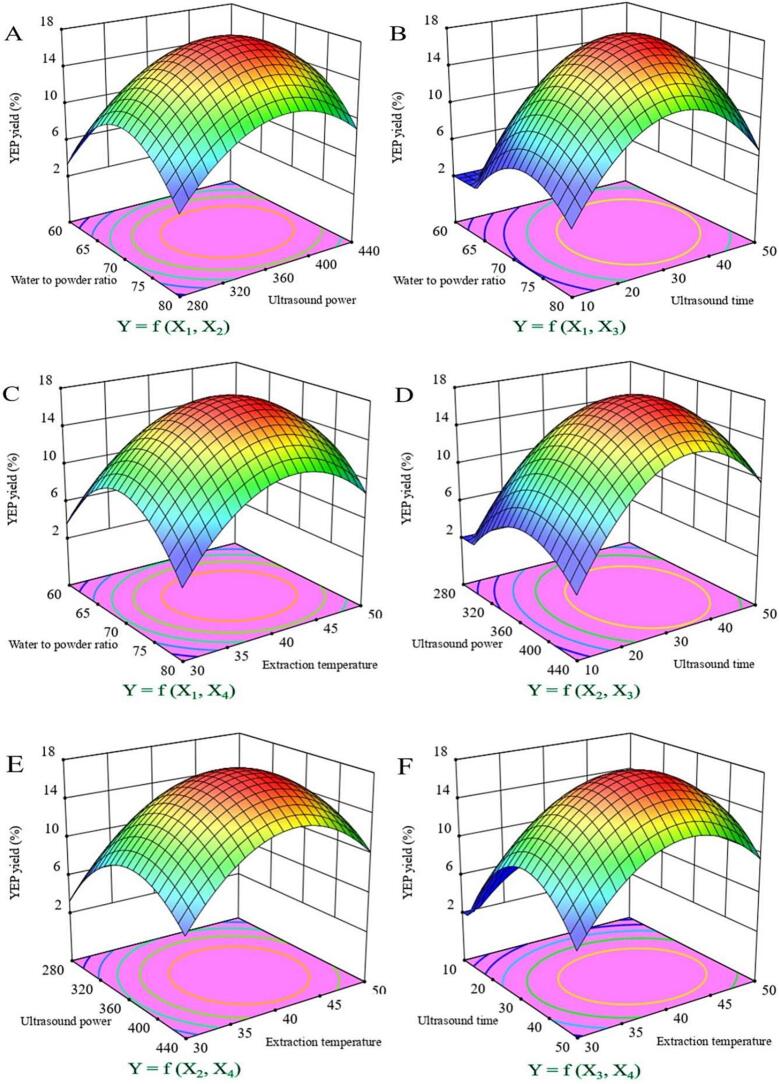
Effects of WPR (X_1_), UP (X_2_), UT (X_3_) and ET (X_4_) on the EYP yield under 90% WBR by response surfaces for optimization of EYP extraction (A - F) from *E. yadongensis*.

In term of three-dimensional (3D) response surface, the optimal conditions for maximal predicted values can be given between two variables [33]. The extraction yield of EYP was apparently related to four variables based on the 3D response surface plots in Fig. 2 (A - F). As shown in the figures, interaction effects between any two factors were visualized intuitively, and the interaction between any two factors showed significantly positive effects on the EYP yield. On the basis of the analysis of response surface contour and the second-order polynomial equation, the optimal conditions of four variables with a predicted yield of 18.15 % of the EYP were the X_1_ 71.8 mL/g, X_2_ 362.4 W, X_3_ 31.2 min and X_4_ 41.5 °C, at WBR 90.0 %.

#### 3.2.3 Extraction validation and efficiency

In order to facilitate the practical operation, the optimal experimental parameters were adjusted as X_1_ 72 mL/g, X_2_ was 362 W, X_3_ 31 min, X_4_ 41.0 °C, and WBR 90.0 %. Based on these parameters, actual extraction yield of 17.92 ± 0.56 % were obtained, which were close to the predicted EYP yield. Thus, the model can be applied to carry out the optimization of EYP extraction from *E. yadongensis*. In this study, the UT and ET were significantly reduced through innovative ultrasound assisted wall-breaking. The advantages of low temperature and minimizing time were evident in the preserved the activity of polysaccharide and improved production efficiency, respectively [34].

we compared MAUE to conventional crushing assisted ultrasound extraction (CAUE), conventional crushing assisted hot water extraction (CHWE) and wall-breaking assisted hot water extraction (BHWE, Table 4). Under similar conditions, MAUE (17.92 ± 0.56 %) gave the highest yield of the EYP than did CAUE (12.55 ± 0.21 %), BHWE (10.38 ± 0.21) and CHWE (7.34 ± 0.13), which indicated that ultrasonic mechanical effects can improve solvent permeation rates, and the breakage of cell walls with mechanical shearing brought high permeability and solubility to strengthen the release of polysaccharide molecules into solvents [35]. In this research, the MAUE was characterized by its high yield, low temperature and short time, making it perfect for the extraction of heat-labile polysaccharide, and then based on the advantages of extraction conditions and yield, the EYP from MAUE method was further studied.

**Table 4:** Comparison of the EYP yields among different methods under the same conditions.

Extraction methods	Extract parameters [13]						EYP yields
	WBR (%)	WPR (mL/g)	particle size (mesh)	UP (W)	UT (°C)	ET (min)	
MAUE	90	72	–	362	41	31	17.92 ± 0.56
CAUE	–	72	160	362	41	31	12.55 ± 0.21
BHWE	90	72	–	–	41	31	10.38 ± 0.21
CHWE	–	72	160	–	41	31	7.34 ± 0.13

### 3.3 Purification and compontent of EYP

#### 3.3.1 Chemical compontent and purification

**Fig. 3:**
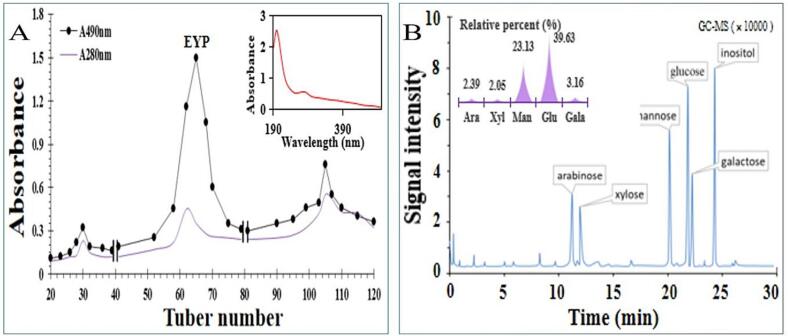
Purification and monosaccharide of the EYP extracted from *E. yadongensis*. A: Elution curve of the EYP; B: GC–MS profile of EYP with acid hydrolysis and acetylation.

The contents of total carbohydrate, moisture, and ash of crude EYP are 93.21 ± 3.54 %, 2.87 ± 0.34 %, and 0.087 ± 0.04 %, respectively, and no protein was detected. The EYP were separated and fractionated (Fig. 3A). As shown in Fig. 3A, the second polysaccharide fraction with relatively higher yield was used to filtrate and elute by Sephadex G-100 gel. The UV spectrum of the EYP in Fig. 3A. Fig. 3A indicated that a slight absorption peak at 280 nm, which indicated the existence of proteins in the EYP, and the protein content was 2.98 ± 0.12 % by Bradford’s assay. Because free protein in the EYP was not detected, the EYP was confirmed as a protein-bound polysaccharide according to the explanation of He et al. [21].

#### 3.3.2 Monosaccharide composition

As shown in Fig. 3B, the EYP was mainly comprised of arabinose, xylose, mannose, glucose and galactose with a relative molar percent of 2.39, 2.05, 23.13, 39.63 and 3.16 %, respectively. Monosaccharide composition of the EYP from *E. yadongensis* was different from the polysaccharides in *Auricularia auricular* [23], which showed that the EYP may be a novel polysaccharide extracted from a new wild edible mushroom *E. yadongensis* in China.

### 3.4 Molecular weight and primary structure of EYP

#### 3.4.1 Molecular weight of EYP

**Fig. 4:**
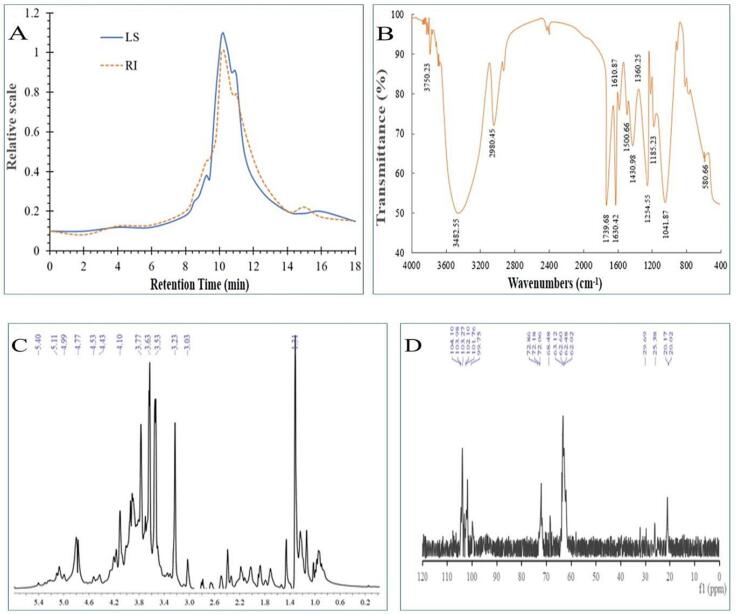
Spectrogram of monosaccharide composition and structural characteristics of EYP from *E. yadongensis*. A: Molecular weight distribution by HPGPC; B: FT-IR spectra and their derivatives; C: ^1^H NMR spectrum; D: ^13^C NMR spectrum.

The *Mw* and *Mn* of EYP were analyzed by HPGPC in Fig. 4A. Fig. 4A indicated that the *Mw* and *Mn* values were calculated as 428.55 kDa and 368.96 kDa, respectively. The PI value of EYP was 1.16, which indicated that the molecular weight distribution of the EYP was relatively narrow. This explained that MAUE may contribute to improve EYP uniformity.

#### 3.4.2 Primary structure of EYP

The structures of the purified EYP, such as functional groups and glucosidic bonds using FT-IR spectrum according to the literatures indicate in Fig. 2B [22]. The comparatively strong absorption peak at 3432.55 cm^−1^ is characterized as O-H, while the band at 2928.78 cm^−1^ is recognized as the C-H. The particular absorption peak at 1650.42 cm^−1^ is characterized as N-H, and the peak at 1420.98 cm^−1^ is characterized as C-O and showed the presence of carboxylate groups. Moreover, the selected polysaccharide has a sharp, strong absorption peak from 1200 to 1000 cm^−1^, which show that a pyranose form of the glucose conformation. The absorption peaks at 938.87–785.88 cm^−1^ was characterized as D-glucopyranose ring and β-D-glucosidic linkages, respectively.

^1^H spectrums of EYP in D_2_O are shown in Fig. 4C. In the range of 4.5–5.5 ppm, EYP had five chemical shifts of heterocephalic hydrogen protons, which indicate that EYP has at least five monosaccharide components. The chemical shifts of the anomeric proton absorption peak at *δ* 4.53, *δ* 4.77 and *δ* 4.99 ppm indicate that EYP containe *β*-glycoside configuration, and absorption peak at *δ* 5.11 and *δ* 5.40 ppm indicate that EYP containe *ɑ*-glycosidic bond configuration. That is, EYP are composed of two glycosidic bonds, *ɑ* and *β*. *δ* 3.03–4.11 ppm are in the region of C2, C3, C4, C5 and C6 protons [36]. The signal at *δ* 1.88 ppm is characterized as N-acetyl group of the protein binding residue [37].

In the ^13^C NMR spectrum of EYP at the same temperature (Fig. 4D), the main signals of the anomeric carbon region of EYP are *δ* 99.75, *δ*101.76, *δ* 102.10, *δ* 103.27, *δ* 103.98, and *δ* 104.10 ppm. Δ 99.75, *δ* 101.76, and *δ* 102.10 ppm are the anomeric carbon signals of ɑ configuration. Δ 103.27, *δ* 103.98, and *δ* 104.10 ppm are the anomeric carbon signals of *β* configuration. The strong signal at *δ* 103.85 ppm matched C-1 of the chain of (1 → 4)-linked *β*-D-glucopyranosyl units (H-1 *δ*4.77 ppm). The low-field intense signal at *δ* 101.77 ppm matched C-1 of the chain of (1 → 6)-linked *ɑ*-D-mannopyranosyl units [21]. The carbon signal at *δ* 72.86–62.02 ppm could be assigned to C2 - C6 of pyranoside ring [38]. Δ 72.06 ppm are the O-O signal peak of the sugar, and there is no signal peak in the range of *δ* 75.00–95.00 ppm, indicating that the sugar was a pyran ring, which is in line with FT-IR spectrum analysis. The peak at *δ* 68.48 ppm correspond to the substituted HO– of *β*-D-mannose [39]. The peak at *δ* 29.69 ppm is attributed to internal acetone. The signal at *δ* 20.02 ppm represented the CH_3_ part of the N-acetyl group, which was the same as ^1^H NMR [40]. Based on the results of the spectroscopy, the main backbone of the EYP is comprised of (1 → 4)-*β*-D-glucopyranosyl and (1 → 6)-*ɑ*-D-mannopyranosyl groups in the main chain.

### 3.5 Bioactivities of EYP

#### 3.5.1 Antioxidant activities

**Fig. 5:**
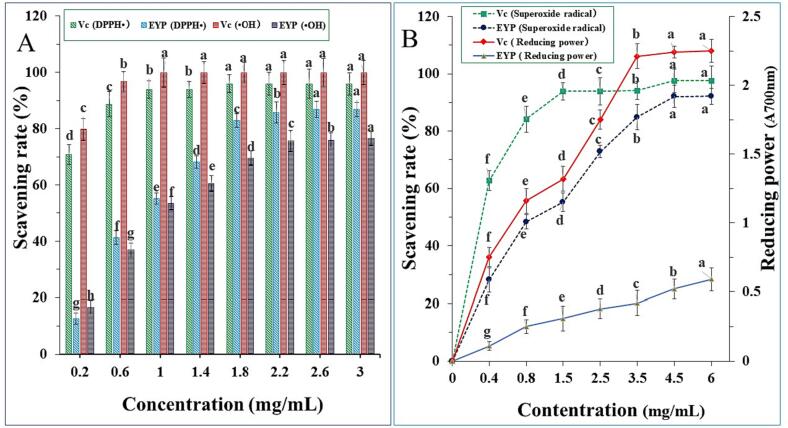
Antioxidant activities of EYP from *E. yadongensis*. A: Scavenging ability on DPPH• and •OH; B: Scavenging ability on superoxide radical and reducing power.

The scavenging rates of the EYP against DPPH• and •OH were indicated in Fig. 5A. The scavenging rates of the EYP against DPPH• radical significantly enhanced along with the increase of the EYP concentrations (*P* < 0.01), which were in line with other study on antioxidant activity of *A. auricula* polysaccharides [23]. When the concentration of the EYP was at 2.6 mg/mL, the DPPH• scavenging rates come to the highest of 86.9 %, indicating that the EYP has strong DPPH• scavenging ability. Similarly, the EYP exhibited definite scavenging activity against •OH radicals (Fig. 5A). Within the scope of 0.2–3.0 mg/mL, the •OH scavenging rates of the EYP were increased with increase of the EYP concentrations. The •OH scavenging rates were less than that of Vc during the same concentration. When the EYP concentration was for 3.0 mg/mL, the •OH scavenging rate was 76.5 %. These results suggested that the EYP would be help to protect against oxidative damage.

The superoxide radical and reducing power of the EYP was indicated in Fig. 5B. The scavenging effects of superoxide radical from the EYP and ascorbic acid were improved with increased concentrations from 0.2 to 4.5 mg/mL. At the concentration of 4.5 mg/mL, the superoxide radical scavenging abilities were 92.13 % and the scavenging activities was significant (*P* < 0.05). The reducing power of the EYP enhanced slowly along with the increasing concentration from 0.2 to 6.0 mg/mL, and the highest reducing power only was 0.59 at the concentration 6.0 mg/mL. A lower reducing power of the EYP as compared to Vc (2.25) was observed, which was similar to the reducing power of JFP-Ps polysaccharides from *Artocarpus heterophyllus* Lam. Pulp [40].

#### 3.5.2 Antibacterial activities

**Fig. 6:**
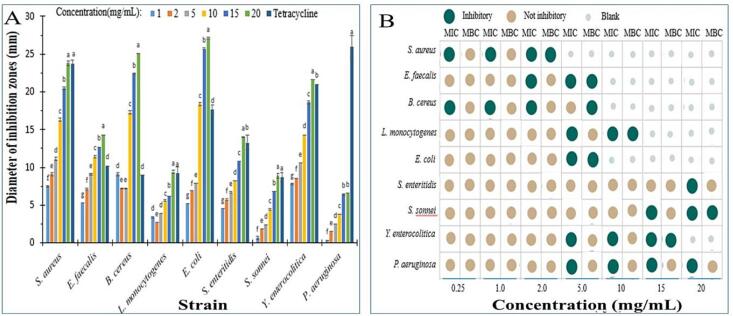
Antibacterial activities of EYP from *E. yadongensis*. A: Diameters of inhibition zones (mm); B: Antibacterial rate as MIC and MBC.

As depicted in Fig. 6A, the EYP displayed different inhibitory activities on all tested strains, and inhibition zones were enhanced with increased concentrations from 0.0 to 20.0 mg/mL. At the concentration of 20.0 mg/mL, the biggest DIZ against *S. aureus*, *E. faecalis*, *B. cereus*, *L. monocytogenes*, *E. coli*, *S. enteritidis*, *S. sonnei*, *Y. enterocolitica*, and *P. aeruginosa* were 23.8, 14.3, 25.1, 9.4, 27.1, 14.0, 8.9, 21.6 and 6.6 mm, respectively. According to DIZ, the EYP showed high antibacterial abilities against all target strains, and obviously higher activity excluding *P. aeruginosa* than commercial tetracycline, highlighting their antibacterial capability. It is been previously reported that hydrocarbons or their oxygenated derivatives display potential antibacterial activities [41]. Thus, reasons for significant antibacterial activity of the EYP from *E. yadongensis* may be due to the presence of these low molecular compounds.

The MIC and MBC values of the EYP were exhibited in Fig. 5B. The results revealed that the MIC and MBC values for all tested bacteria were at the 0.25 to 20.0 mg/mL and 2.0 to 20.0 mg/mL range, respectively. Surprising, the MIC values of the EYP for all tested bacteria have been obtained when the EYP reached the maximal concentration in tested range. However, the MBC values of the EYP for *S. enteritidis* and *P. aeruginosa* have not been gained in tested range. Among all bacteria, the EYP indicated both a minimum MIC of 0.25 mg/mL and MBC of 2.0 mg/mL against *S. aureus* and *B. cereus*, which showed the EYP may become an ideal effective bacterial inhibitor. Based on these findings, gram positive bacteria like *S. aureus* and *B. cereus*, are thought to be more susceptible to the EYP inhibitory rate, which was different from the other study that showed gram positive bacteria like *E. coli* and *K. pneumoniae* were more susceptible to mushrooms inhibitory rate [42]. Therefore, the inhibitory mechanism of action of the EYP against *S. aureus* and *B. cer*eus will be investigated in the future.

#### 3.5.3 Antidiabetic and antitumor activities

It is advantageous for diabetics to inhibit the hydrolytic abilities of α-amylase and α-glucosidase. Table 4 showed that EYP had significant inhibition abilities against both α-amylase and α-glucosidase, and the inhibition activities increased obviously with an increase of EYP concentration from 50.0 to 150.0 μg/mL (*P* < 0.05). When the EYP concentration increased further, the inhibition abilities declined slowly. The highest inhibition activities were obtained at 150 μg/mL, and were 91.3 % and 89.8 %, respectively. Similarly, EYP had significant antitumor abilities against colon and breast carcinoma cell lines (Table 5). The highest antitumor activities against Caco-2 and MCF-7 were 49.44 % and 42.11 % at 10.0 and 12.5 mg/mL, respectively. The antidiabetic and antitumor ablities of EYP are closed to those achieved by other polysaccharide (EPS) [27]. Some mechanisms have been reported to explain the antitumor abilities of different polysaccharides [43]. Similarly, the mechanism of the antidiabetic and antitumor activities of the EYP will be investigated in the future.

**Table 5:** Antidiabetic and antitumor activities of EYP from *E. yadongensis.*

Attributes	Bioactivities of EYP					
Antidiabetic activity	EYP concentration gradients (μg/mL)					
	50	100	150	200	250	300
⍺-amylase inhibition (%)	78.90 ± 0.67^c^	90.00 ± 0.56^a^	91.30 ± 0.52^a^	90.80 ± 0.53^a^	90.40 ± 0.61^a^	89.80 ± 0.62^b^
⍺-glucosidase inhibition (%)	69.80 ± 0.88^d^	88.50 ± 0.67^b^	89.80 ± 0.54^a^	87.90 ± 0.57^b^	87.80 ± 0.43^b^	87.20 ± 0.52^c^
Antitumor activity	EYP concentration gradients (mg/mL)					
	2.5	5	7.5	10	12.5	15
Colon cancer cell line Caco-2 (%)	12.50 ± 0.98^d^	32.50 ± 0.67^c^	40.25 ± 0.66^b^	49.44 ± 0.73^a^	48.60 ± 0.56^a^	48.22 ± 0.67^a^
Breast cancer cell line MCF-7 (%)	10.020 ± 0.98^d^	27.23 ± 1.34 ^cd^	38.67 ± 0.76^bc^	41.56 ± 0.56^abc^	42.11 ± 0.54^abc^	41.88 ± 0.44^ab^

The biological activities of polysaccharide are closely related to its structural characteristics, and the structural differences depends on the extraction method or different temperature [44]. The effect of temperature was reported: the polysaccharide extracted at 30 °C indicated stronger bioactivities than that at 90 °C from mulberry fruit [45]. These researches revealed that high extraction temperature can lead to polysaccharide degradation, resulting in a decrease in bioactivities [46]. Additionally, polysaccharides with high content had the advantage of quantity for the bioactivity exhibition [47]. As far as current research is concerned, EYP extracted by MAUE exposed stronger bioactivities than that extracted by other ultrasonic extraction technology [16], [17].

### 3.6 Rheological behavior of different concentration and pH of EYP solutions

The rheological property of the EYP is an important feature that appeals to the yogurt and other food industries. Therefore, the effect of various conditions, such as the concentration and pH of EYP solutions on the rheological properties of EYP should be researched.

#### 3.6.1 Apparent viscosity and shear stress

**Fig. 7:**
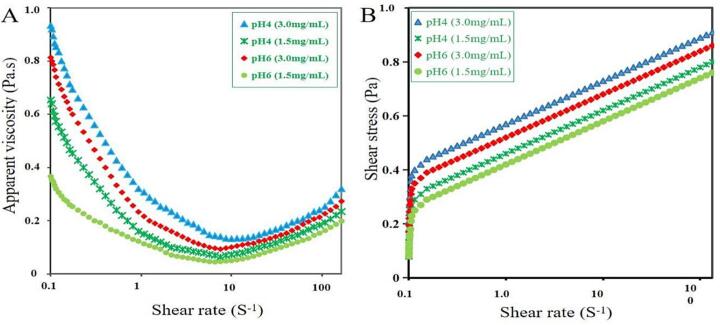
Apparent viscosity (A) and shear stress (B) of different concentration and pH of EYP solutions.

Fig. 7A illustrated that the apparent viscosity of EYP solutions at different shear rates. The apparent viscosity of the EYP solutions decreased significantly with increasing shear rate from 1 to 100 s^−1^ (*P* < 0.05), regardless of different concentration and pH value, which suggested that EYP solutions presented a non Newtonian shear-thinning behavior [48]. As seen in Fig. 4A, there were obvious differences in the apparent viscosity of EYP solutions with various concentrations and pH values (*P* < 0.05). At a shear rate of 1 s^−1^, EYP aqueous solutions of EYP_3.0_-pH4 (3.0 mg/mL, pH4) demonstrated the highest apparent viscosity, followed by EYP_3.0_-pH6 (3.0 mg/mL, pH6), EYP_1.5_-pH4 (1.5 mg/mL, pH4) and EYP_1.5_-pH6 (1.5 mg/mL, pH6). This outcome implied that high concentration and low pH value of EYP solutions had a positive impact on the apparent viscosity of EYP product, which is a technical advantage in acid food industry, such as yogurt. These behaviors of EYP solutions may be helpful to the inter molecular bridges built by changes of concentration and pH value in EYP solutions [49].

The data of power law model of R^2^ > 0.997 fitted well all flow curves of EYP solutions under both concentration and pH values (Fig. 7B). EYP solutions prepared with various concentrations and pH values had different deviation from Newtonian behavior. Consistency coefficient values (m) were EYP_1.5_-pH6 > EYP_1.5_-pH4 > EYP_3.0_-pH6 > EYP_3.0_-pH4, and the values of flow behavior indices (n) were EYP_3.0_-pH4 > EYP_3.0_-pH6 > EYP_1.5_-pH4 > EYP_1.5_-pH6. Generally, four EYP solutions have minor K values and bigger n values, showing that their pseudoplasticity is less pronounced. The well-fitting model of EYP solutions provided evidence showed that the pseudoplasticity of EYP product is an important feature in yogurt or other food production.

#### 3.6.2 Flow curves

**Fig. 8:**
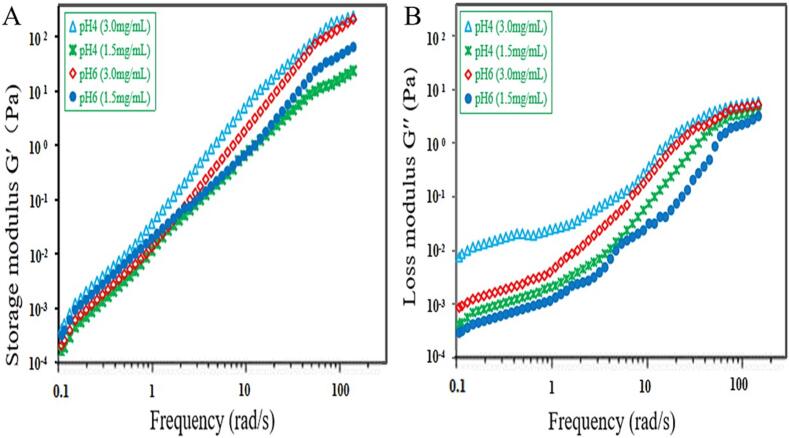
Storage modulus (G′, A) and loss modulus (G′′, B) curves of different concentration and pH of EYP solutions.

The frequency sweep tests were carried out measure the linear range of the storage (G′, Fig. 8A) and loss (G′′, Fig. 8B) moduli. The results indicated that the G′ and G′′ values of all EYP solutions improved along with frequency and exhibited a significant frequency dependence in the range of texted frequency, which indicated that the EYP product had a more viscous behavior than elastic behavior. The viscoelastic features of the EYP exhibited a better tolerance towards the change in the concentrations and pH of EYP solutions, which was in line with those researched by Han et al. [49]. For four EYP solutions, the loss factor indicated that G′ < G′′, in the frequency range of 1.0–5.0 Hz. Then, the storage modulus (G′) is dominant over the loss modulus (G′′) at f > 5.0 Hz. The change of G′ and G′′ of EYP product contradicted the polysaccharide produced from *Sporidiobolus pararoseus* jd-2 [49].

In general, the decrease in pH can lead to a weakening of viscosity of the polysaccharide, which can be due to strong acid hydrolysis under low acidic pH [50]. However, the viscosity of the EYP aqueous solution increased at lower pH, which may be related to that the broken wall of the *E. yadongensis* caused the number of molecular links smaller and the link length shorter of the EYP, and the ultrasonic cavitation effects produced high solubility to the EYP [51]. Additionally, EYP was confirmed as a protein-bound polysaccharide, and it is well known that proteins are very sensitive to pH, which may enhance the interaction of the intermolecular hydrogen bonds between EYP and solvents [52], and as a result, the viscosity was increased.

## 4 Conclusion

Ultrasound assisted mechanical wall-breaking extraction (MAUE) was successfully established for the EYP extraction from a new *E. yadongensis*. Based on the MAUE with RSM, the polysaccharide yield of 17.92 ± 0.56 % with the optimal parameters of five extraction factors were obtained, and current MAUE was characterized by its high yield, low extraction temperature and short ultrasound time. After the isolation and purification, EYP (Mw: 428.55 kDa) was comprised of Arabinose, Xylose, Mannose, Gglucose and Ggalactose with a relative molar percent of 2.39, 2.05, 23.13, 39.63 and 3.16 %, respectively. FT-IR and NMR analysis showed that the main backbone of EYP comprised of (1 → 4)-*β*-D-glucopyranosyl and (1 → 6)-*ɑ*-D-mannopyranosyl groups. The EYP had significant DPPH• (86.9 %), •OH radical scavenging (76.5 %), and superoxide radical scavenging (92.1 %) effects, respectively (*P* < 0.05), and exhibited amazing antibacterial activities against all tested strains. Among these tested bacteria, *S. aureus* and *B. cereus* indicated the lowest MIC and MBC values of 0.25 and 2.0 mg/mL, respectively. Moreover, the EYP had effective inhibition ablities against both α-amylase (90.8 %) and α-glucosidase (89.8 %), and antitumor activities against colon (49.4 %) and breast carcinoma cell lines (42.1 %), and EYP product exhibited good viscoelastic properties and tolerance in low pH solutions.

## CRediT authorship contribution statement

**Ying Tang:** Validation, Writing – original draft. **Yuzhi Miao:** Project administration, Formal analysis, Software, Formal analysis, Writing – review & editing. **Min Tan:** Validation. **Qinqin Ma:** Data curation, Investigation, Resources, Methodology, Funding acquisition, Supervision. **Chengyi Liu:** Formal analysis, Writing – review & editing. **Mei Yang:** Investigation, Resources, Methodology. **Yanqiu Su:** Validation. **Qi Li:** Conceptualization, Project administration.

## Declaration of Competing Interest

The authors declare that they have no known competing financial interests or personal relationships that could have appeared to influence the work reported in this paper.
