# Supplementary material for: Ultrasound assisted wall-breaking extraction and primary structures, bioactivities, rheological properties of novel Exidia yadongensis polysaccharide
Source: Ultrason Sonochem. 2023 Oct 30;101:106643. doi: 10.1016/j.ultsonch.2023.106643 (PMC10641719; doi:10.1016/j.ultsonch.2023.106643)
Supplement: Supplementary data 1 [file mmc1.docx]

Table S1 a central composite design (CCD) with four factors including WPR (X_1_), UP (X_2_), UT (X_3_) and ET (X_4_).

| Factors | Coded (actual) variables | | | | |
| --- | --- | --- | --- | --- | --- |
|  | -2 | -1 | 0 | 1 | 2 |
| X_1_ (mL/g) | 60 | 65 | 70 | 75 | 80 |
| X_2_ (W) | 280 | 320 | 360 | 400 | 440 |
| X_3_ (min) | 10 | 20 | 30 | 40 | 50 |
| X_4_ (°C) | 30 | 35 | 40 | 45 | 50 |
